# Melanosis of the Bladder: Possible Pathogenetic Mechanisms

**DOI:** 10.1155/2022/6221499

**Published:** 2022-06-16

**Authors:** Justin E. Dupey, Sarah J. Wood, Richard Y. Ball

**Affiliations:** ^1^Norfolk and Waveney Cellular Pathology Service, Norfolk and Norwich University Hospitals NHS Foundation Trust, Colney Lane, Norwich, Norfolk NR4 7UY, UK; ^2^Department of Urology, Norfolk and Norwich University Hospitals NHS Foundation Trust, Colney Lane, Norwich, Norfolk NR4 7UY, UK

## Abstract

Melanin accumulation within the bladder urothelium and/or macrophages in the lamina propria (melanosis of the bladder) is a very rare phenomenon of unknown pathogenesis. Its rarity argues for a complex, likely multifactorial, causation. We describe bladder melanosis developing after Botox therapy in an elderly woman with a history of overactive bladder, treated grade 2 uterovaginal prolapse, and episodes of urinary tract infection and speculate that one factor (probably of many) in its pathogenesis may be a derangement of local neurourothelial interactions.

## 1. Introduction

Melanosis of the bladder is the presence of melanin within the bladder urothelium and/or macrophages in the lamina propria. It was first recorded in humans in 1986 [1], since when about two dozen additional cases have been reported [2–21]. It affects middle-aged and older men and women (age range 43–86 years; median age = 65 years, including our case), with a slight male preponderance (M : F = 1.5). It is usually discovered when patients undergo cystoscopy to investigate various non-specific urinary tract symptoms. The pathogenesis is obscure.

In this report, we describe a case developing after botulinum toxin (Botox) therapy for an overactive bladder and discuss possible pathogenetic mechanisms.

## 2. Case Presentation

In September 2014, a 69-year-old woman presented to a gynaecologist with a history of urgency, urge incontinence, nocturia ×3–4, and occasional voiding difficulty. She had grade 2 uterovaginal prolapse on straining. A cystocoele, extending almost to the level of the hymen, was present. Subsequent urodynamic studies showed detrusor overactivity with minimal stress incontinence. She was advised to start anticholinergics for bladder overactivity and was counselled for surgery for the uterine prolapse. In October 2015, she underwent vaginal hysterectomy. Six months later, she complained of urgency, urge incontinence, and urine leakage on bending and standing. Gynaecological examination showed a well-supported wall and minimal cystocoele. Her symptoms continued, despite changing to mirabegron, and Botox therapy was discussed. She was taught how to undertake intermittent self-catheterisation (ISC) prior to Botox therapy. A urinary tract infection (UTI) was noted at first presentation for Botox injections, and the procedure was deferred.

In April 2017, cystoscopy under general anaesthesia (GA) revealed a normal urothelium; 100 units of Botox were injected into 10 sites in the bladder, sparing the trigone. At review two months later, her symptoms were no better. She had not attempted ISC postoperatively, and a residual urine volume at that time was 300 mL. She was again encouraged to perform SC twice daily to drain the residual urine.

Six months post-Botox, she continued to be symptomatic. She had not performed ISC (residual urine volume still 300 mL) and had problems with recurrent UTIs. Her infections were treated, and modifications in medication were tried to control her bladder overactivity.

**Figure 1:**
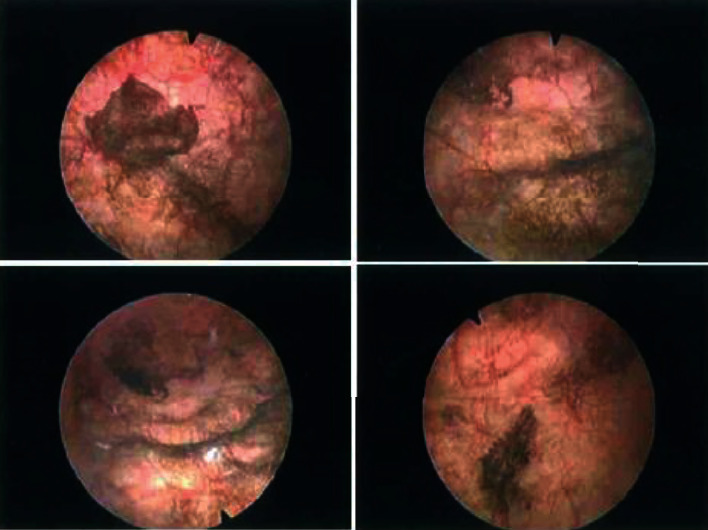
Cystoscopic images showing patches of melanosis of the bladder.

In September 2018, she was referred to the Urology Department because of persistent nonvisible haematuria. She stated that her urinary symptoms, particularly urgency and urge incontinence associated with hesitancy on voiding, had not been controlled. Repeated MSUs had been positive despite being asymptomatic for infection. Ultrasound scan showed normal upper tracts and a postvoid residual urine volume of 260 mL. Flexible cystoscopy and subsequent GA cystoscopy revealed a normal urethra and a large-capacity bladder with dark-staining patches throughout (Figure 1), but sparing the trigone; there was no evidence of tumour. Four cold cup biopsies were obtained and cystodiathermy was undertaken.

She continued to have UTIs. Follow-up flexible cystoscopy 10 months after GA cystoscopy showed ongoing areas of pigmentation (which were possibly less severe than previously). She was started on low-dose antibiotics and was again advised to perform ISC.

In January 2020, because of ongoing infections, she was prescribed intravesical iAluRil weekly instillation for 4 weeks and further monthly single doses for the following 5 months, totalling 9 doses. She would sporadically perform ISC last thing at night only. This regime was interrupted because of the COVID-19 lockdown, and she had further problems with infections and incontinence.

In September 2020, cystoscopy showed patches of pigmentation dotted throughout the bladder, but they did not seem as marked as previously. She was again advised to increase ISC to twice daily (150 mL drained bd) and to recommence iAluRil instillations.

## 3. Histopathology Findings

**Figure 2:**
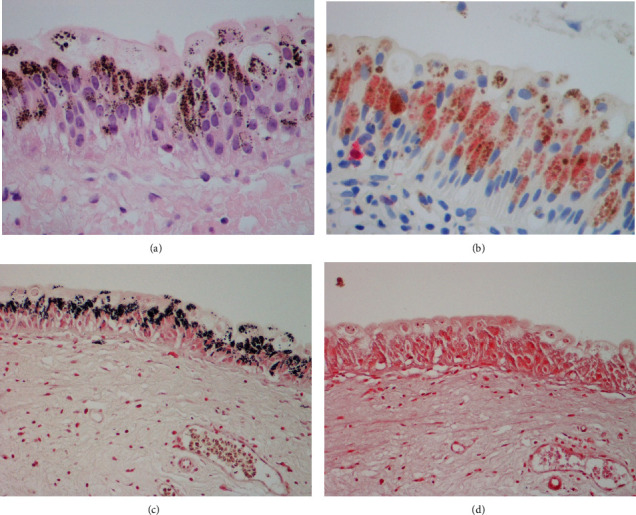
Histopathological preparations of bladder mucosa showing melanosis. (a) H&E-stained section showing melanin in the urothelium. (b) Many of the pigmented urothelial cells are immunoreactive for S100 protein (red stain). (c) The melanin is shown (black) in a Masson Fontana preparation. (d) The Masson Fontana reaction is abolished by prior treatment of the section with a bleach.

The biopsies showed widespread dark brown cytoplasmic pigment in the urothelium, present at many levels but most abundant in the outer layers, especially the umbrella cells (Figure 2). It consisted of small to medium-sized granules with occasional course lumps and was positive with the Masson Fontana method (Figure 2). That reaction was abolished by prior treatment of the section with a bleach, confirming melanin and establishing the diagnosis of melanosis. The pigment was not ceroid/lipofuscin (negative with the Ziehl-Neelsen (ZN), modified ZN, oil-red-O and PAS.D methods) or haemosiderin (negative with Perls' method).

Immunohistochemical staining showed that the urothelium, including that containing pigment, was GATA-3-positive. Many (but not all) of the pigmented urothelial cells stained moderately strongly for S100 protein (Figure 2) and weakly for CD68. No definite melanocytes were recognised within the urothelium. There was no evidence of dysplasia or malignancy.

The superficial lamina propria showed focal mild nonspecific chronic inflammation. Granules of melanin were present in a few macrophages (CD68+).

## 4. Discussion

Bladder melanosis has been reported in patients with a variety of symptoms and findings, including: urinary obstruction, urgency, and incontinence; lower urinary tract symptoms; flank or loin pain; haematuria; and UTIs. It may be a transient phenomenon: one report [19] indicates that melanosis may completely resolve spontaneously within a year. It has also resolved after BCG therapy for urothelial carcinoma [8]. Our patient, an elderly woman with an overactive bladder, treated grade 2 uterovaginal prolapse and subsequent episodes of UTI, resembled in many respects the typical patient with bladder melanosis. Her melanosis developed after a prolonged sequence of lower urinary tract symptoms associated with detrusor overactivity (followed by Botox therapy) and recurrent UTIs. Cystoscopy at the administration of Botox showed normal urothelium. While it may be tempting to believe that there was a causal link between the Botox therapy and the development of bladder melanosis, that association may be coincidental. The melanosis could perhaps have been caused by her UTIs. Such an association has been reported previously [8, 12].

Melanocytes are not a normal component of bladder urothelium. When discussing the origin of bladder melanosis, two main mechanisms, first mentioned by Rossen and Petersen [2], have been put forward to explain the assumed acquisition of melanocytes by the urothelium: migration of neural crest cells or their differentiation from local stem cells. However, very few reports indicate the presence of melanocytes in the urothelium, raising doubt about the validity of these proposals. However, it is possible that the acquisition of melanocytes by the urothelium is transient. A third possible process, melanuria, was mentioned by Pandian and colleagues [15], who noted that the melanin appeared to be within lysosomes, rather than melanosomes. If that were the case, melanosis of the bladder might be commoner in dark-skinned races than in white Caucasians. The race of the subjects is described in a minority of the case reports; where noted, Caucasians represent 80% and Afro-Caribbeans 20% of the subjects. While it cannot be excluded, melanuria seems an unlikely explanation.

If bladder melanosis is not the result of migration of neural crest cells, transdifferentiation of urothelial stem cells, or melanuria, how does it occur? Two other potential mechanisms might be considered: an abnormal reaction of the mucosal nerve plexus and the plasticity of urothelium. Our patient's melanosis developed after Botox therapy, which draws attention to the possible significance of the innervation of the bladder in the pathogenesis of melanosis. Some of the reported patients also had symptoms suggesting overactivity of the bladder or of outflow obstruction, conditions that would be expected to be associated with local neural and neuromuscular dysfunction.

The urothelium is more than a physical barrier: it has specialised sensory and transduction or signalling properties [22]. The expression of various receptors and ion channels implies that the urothelium can respond to a variety of stimuli. Nerve fibres are in close proximity to the urothelium. There may, therefore, be reciprocal communication between urothelial cells and nearby nerves and other cells types, such as smooth muscle cells and interstitial cells of Cajal [22].

Urothelium, both in its native state and when neoplastic, is recognised to have the potential to differentiate along various lines, often in a focal and patchy manner. The urothelium in our case showed focal staining of the pigmented urothelial cells (GATA-3-immunoreactive) for S100 protein, suggesting that they may have developed some neural or melanocytic phenotypic features. Might that imply their acquired ability to synthesise melanin? Alternatively, it is possible that melanin may have been produced focally within nerve cells in the superficial lamina propria and then transferred to the urothelium, rather in the manner of melanocytes and keratinocytes in the skin.

A unique case of melanosis of the bladder has been reported in a 7-year-old cow [23]. Melanin was present within the lamina propria in melanocytes and melanophages but was not seen in the urothelium. Bovine papilloma virus type 2 (BPV-2) DNA was detected, and there was evidence of mild chronic inflammation in the lamina propria. The authors suggested that the mild interstitial cystitis was related to BPV-2 infection but not to the melanosis. Melanophages in the lamina propria of the bladder have also been described in published human cases [3, 6, 8, 9, 14–16, 20, 21], as well as in our case. However, the bovine case was pathologically different from those so far reported in humans and the pathogenetic mechanisms may be different.

## 5. Conclusion

Melanosis of the bladder appears to be a rare acquired, sometimes transient, state. Its pathogenesis remains obscure. Given its rarity, the cause is likely to be complex and multifactorial; it may well be an idiosyncratic reaction. Published cases suggest the possibility that derangement of neural processes in the bladder wall and their interaction with the urothelium may be important. In our patient, an overactive bladder with episodes of UTI may have been significant underlying factors in pathogenesis, possibly exacerbated by the effects of Botox therapy.

## Data Availability

Data as such are not part of the paper. The literature we used is listed in the manuscript.
